# Pharma-Nutritional Properties of Olive Oil Phenols. Transfer of New Findings to Human Nutrition

**DOI:** 10.3390/foods7060090

**Published:** 2018-06-11

**Authors:** M. Carmen Crespo, Joao Tomé-Carneiro, Alberto Dávalos, Francesco Visioli

**Affiliations:** 1 Laboratory of Functional Foods, Madrid Institute for Advanced Studies (IMDEA)-Food, CEI UAM + CSIC, 28049 Madrid, Spain; carmen.crespo@imdea.org (M.C.C.); joao.estevao@imdea.org (J.T.-C.); 2 Laboratory of Epigenetics of Lipid Metabolism, Madrid Institute for Advanced Studies (IMDEA)-Food, CEI UAM + CSIC, 28049 Madrid, Spain; alberto.davalos@imdea.org; 3 Department of Molecular Medicine, University of Padova, Viale G. Colombo 3, 35121 Padova, Italy

**Keywords:** olive oil, biophenols, Mediterranean diet, pharma-nutrition, cardiovascular disease

## Abstract

The Mediterranean diet has been long associated with improved cardiovascular prognosis, chemoprevention, and lower incidence of neurodegeneration. Of the multiple components of this diet, olive oil stands out because its use has historically been limited to the Mediterranean basin. The health benefits of olive oil and some of its components are being rapidly decoded. In this paper we review the most recent pharma-nutritional investigations on olive oil biophenols and their health effects, chiefly focusing on recent findings that elucidate their molecular mechanisms of action.

## 1. Introduction

Adherence to a Mediterranean-style diet has long been associated with improved cardiovascular prognosis, chemoprevention, and lower incidence of neurodegeneration [1]. Mediterranean diets are quite variegated in composition, but share some common traits, as outlined by Martínez-González et al. [2]. Of the multiple components of the Mediterranean diet, the use of olive oil as a principal source of fat stands out because it is characteristic of the Mediterranean basin [3]. Indeed, in places like Crete, fat consumption reaches 40% of total calories, yet nearly all of this comes from olive oil.

Historically, olive oil has been attributed religious characteristics and has also been used for cosmetic purposes [4]; its culinary/alimentary use has been overlooked until relatively recent times. Research on the biological properties of olive oil is even more recent and can be traced back to 1994, with the first publication reporting inhibition of low density lipoprotein oxidation by oleuropein (OLE), the bitter principle of olives [5]. It is noteworthy that this research was triggered by a publication authored by Papadopoulos et al. [6], where the authors indicated hydroxytyrosol (HT) as indispensable for olive oil stability.

When it comes to food and its components, it is incorrect to talk about pharmacology and pharmacological activities. Pharmacology follows obligatory pathways that bring a drug to the market. Of note, drugs have measurable effects on the human body, whereas foods and their components are necessarily weaker in their actions [7]. The area in which dietary molecules are being studied is called pharma-nutrition, in that it transcends pure nutrition (calories, macronutrient proportions, etc.), yet does not concern therapy and purely medicinal actions.

In this paper we briefly review pharma-nutritional evidence from the last decade that indicates how extra virgin olive oil (EVOO) components might exert important physiological actions that bring about cardioprotection, chemoprevention, and prevention of neurodegenerative processes. Then, as several other reviews are available (e.g., [3,8]), we focus on the latest findings addressing molecular mechanisms of action.

## 2. Pharma-Nutritional Actions: A Summary of Recent Evidence

### 2.1. Cardioprotection

Most pharma-nutritional studies with olive oil biophenols are being carried out in the cardiovascular arena (note that we will use the term “biophenols” throughout the text because extra virgin olive oil contains a large variety of molecules, many of which are non-phenolic in nature). In vitro experiments with pure HT started 25 years ago [9] and led to the European Food Safety Authority (EFSA, Parma, Italy) granting HT a (somewhat debated) health claim based on this activity [10]. This is—in part—the result of many animal and human studies that have been performed in various experimental conditions; the vast majority indicate that olive oil biophenols do modulate a variety of surrogate markers of cardiovascular disease (CVD) [3]. We discuss the molecular actions below, but it is worth underscoring that investigation on the healthful potential of olive oil biophenols, namely HT, is very advanced and includes nutrigenomic [11] and proteomic studies [12] (vide infra). In terms of surrogate marker modulation, the effects of HT on cholesterol concentrations are apparently modest, yet other risk factors of CVD are positively modulated by olive biophenols [3]. One lipid-related example is that of HDL particles, for which functionality is improved by EVOO biophenols [13].

### 2.2. Chemprevention of Cancer

With regard to chemoprevention of cancer, the situation is fairly complex in that animal models and surrogate markers in humans are scant and impede firm conclusions from being drawn [14]. However, epidemiological studies consistently report an inverse association between adherence to the Mediterranean diet and incidence of breast cancer [15]. This association is stronger for postmenopausal breast cancer prevention [16].

In this respect, targeting inflammation as one of the major players in tumor incidence and recurrence appears to be a sensible strategy [17]. As mentioned above, olive biophenols have anti-inflammatory activities and might play protective roles in this area [18]. Also, an increase in nucleophilic tone would contribute toward chemoprevention and accelerated recovery from cancer, as shown for e.g., curcumin [19].

In addition to inflammation, some mechanistic studies have been performed to explain the potential preventive actions of olive oil biophenols on cancer. Mechanisms of action might include inhibition of cell proliferation and tumor progression as well as increased rates of apoptosis (see for example [20,21]).

Finally, it is worth mentioning that a secondary analysis from the Prevention with Mediterranean Diet (PREDIMED) study assessed the effect of a dietary intervention encouraging the adherence to a Mediterranean diet on the incidence of postmenopausal breast cancer among 4152 women aged from 60 to 80 years of age [22]. The results showed that women who consumed at least 15% of EVOO in terms of total energy intake exhibited a significant reduction in breast cancer risk when compared to women for which extra-virgin olive oil consumption was lower than 5% of total caloric intake. Whether the preventive effects of olive oil are due to its biophenols or to other unknown confounders is a matter for further investigation.

### 2.3. Neurodegeneration

One of the major challenges of current public health policy is the increasing prevalence of mental illness and neurodegenerative diseases, which is largely due to the rapid aging of the Western, i.e., European and American population. In socio-economic terms, this phenomenon is placing a heavy burden on national health care systems and on the overall population. Preventive strategies are indispensable and the most effective one is the early adoption of a healthy lifestyle and appropriate diet.

Epidemiological studies [23,24] have consistently associated olive oil consumption with better cognition. Moreover, several meta-analyses of observational studies suggest that using olive oil as the main culinary fat can reduce the incidence of depression [25,26]. Even though these association might be casual, some ad hoc studies with olive biophenols are being undertaken. One example is that of HT, which was able to restore proper insulin signaling in an in vitro model of Alzheimer’s disease (AD) [27]. It is also noteworthy that Qosa et al. tested the effects of EVOO [28] and of oleocanthal (OC) [29] in a transgenic mouse model of AD. They reported lower beta-amyloid deposition, which corroborates the scant in vitro data available thus far.

### 2.4. Absorption, Distribution, Metabolism, and Elimination (ADME)

As in traditional pharmacology, pharma-nutrition studies gain credibility and strength when they assess and elucidate absorption, distribution, metabolism, and elimination of the putative active compound. In the case of olive oil and its phenolic components, the first evidence of human absorption was published in the year 2000 [30]. At the time, there were no available techniques to evaluate plasma concentrations of biophenols. Therefore, only urinary metabolites were measured. Subsequent studies confirmed and expanded those findings [31]. To date, the most comprehensive and technologically-advanced study is that of Pastor et al. [32]. In that study, the authors report C_max_ of HT of 2.8 10^−6^ mol/L, following ingestion of EVOO. HT excretion can also be evaluated after the administration of an olive mill waste water (OMWW) preparation devoid of secoroidoids. Khymenets et al. measured HT urinary concentrations and reported HT-S-3’ as the major metabolite [33]. Of note, Gonzalez-Santiago et al. [34] described the association of HT to LDL after intake of the pure molecule. This might be important in light of the purported activities of HT in reducing ox-LDL concentrations, as per the EFSA health claim.

In short, there is plenty of information available on the ADME of HT in humans and rats. Of note, D’Angelo et al. had access to tritiated HT and reported its accumulation in the rat brain (the only such piece of evidence thus far) [35]. In summary, accumulated research indicates the low bioavailability of HT (common to nearly all biophenols) and therefore, strategies are in place to create formulations to overcome this issue.

### 2.5. Toxicity

EVOO consumption—of course—is safe and the only drawback of excessive use is heightened caloric intake. In light of the use of olive biophenols as nutraceuticals or functional foods ingredients, international bodies require proof of absence of toxicity. HT has been tested in a variety of models and a NOAEL of 500 mg/kg/d has been proposed [36,37]. The recent Novel Food (NF) status granted to HT outlines that “Taking into account that the anticipated daily intake of the NF would be in the range of or even less than the exposure of HT from the consumption of olive oils and olives, which has not been associated with adverse effects, and considering the similar kinetics of HT in rats and humans, […..] the Margin of Exposure for the NF at the intended uses and use levels is sufficient for the target population. The EFSA Panel concludes that the novel food, HT, is safe under the proposed uses and use levels” [38]. Finally, HT is generally recognized as safe (GRAS) in the USA and, in summary, there is no clear evidence of toxicity even at high doses.

In any event, caution should be exerted when using any kind of supplements/functional foods in the absence of clear health benefits and as a replacement for a healthful and balanced diet.

## 3. Molecular Insights into Mechanisms of Action

Reactive oxygen species (ROS) and reactive nitrogen species (RNS) are important inflammatory effectors contributing to the elimination of invading pathogens and supporting tissue repair, accelerating the resolution of inflammation. However, ROS/RNS can trigger the generation of inflammatory initiators (e.g., inflammatory cytokines) and damage macromolecules such as lipids, proteins, and nucleic acids. This damage eventually leads to cell death and tissue deterioration [39], which stimulates the development of several diseases, including those of a neurodegenerative nature [40], atherosclerosis [41], metabolic syndrome (MS) [42], type 2 diabetes (T2DM) [43], liver diseases [44], and cancer [45].

Numerous studies performed with animal and cell models suggest that biophenol intake may be beneficial for the prevention and adjuvant treatment of such diseases [46]. In particular, olive oil and its phenolic compounds exert beneficial health effects that encompass anti-inflammatory and antioxidant (direct or indirect) mechanisms, as reflected in many reviews [47,48,49,50,51]. We will briefly review recent evidence arising from studies carried out in the most recent decade (especially in the last lustrum), pointing to the protective effects of olive oil and its phenolic compounds in the context of neurodegenerative disease, CVD, liver disease, cancer, and rheumatic disease.

## 4. Cardiovascular Disease, Metabolic Syndrome, Type 2 Diabetes

A possible link between inflammation, endothelial dysfunction, and CVD is increased oxidative stress (now called redox code [52]) [53]. Inflammation participates in atherosclerosis from its inception and development to its ultimate endpoint, thrombotic complications. Oxidative stress has been identified as critical in most of the key steps in the pathophysiology of atherosclerosis [54]. Endothelial dysfunction involves deviations in the regulation of vascular tone and vascular smooth muscle growth, monocyte adhesion, platelet function, and fibrinolytic activity, which are critical in the development and progression of atherosclerosis and its complications. Reduction of nitric oxide (NO) availability is a main alteration responsible for endothelial dysfunction [55]. Regular consumption of high-fat and high-carbohydrate diets promote increased oxidative stress and inflammation that can result in a host of inter-related metabolic abnormalities and endothelial dysfunction [56,57].

In vitro, EVOO phenolic-rich extracts counteract oxidative stress. They decrease ROS production and levels of malondialdehyde (MDA) [58], downregulate inducible nitric oxide synthase (iNOS) and cyclooxygenase 2 (COX-2) expression, reduce MAPK (JNK, p38) phosphorylation and nuclear factor κB (NF-κB) translocation [59,60], and reduce VEGF-induced angiogenic responses by preventing endothelial NADPH oxidase activity [61]. They also decrease the expression of selective NADPH oxidase subunits. In rat hearts, diet supplementation with oil or oil products containing EVOO-polar biophenols attenuated a hypercholesterolemia-induced increase in MDA and TNF-α [62], and HT administration improved doxorubicin-enhanced cardiac disturbances, probably by affecting the mitochondrial electron transport chain [63]. Regarding human studies, in healthy subjects, supplementation with olive oil, either low or high in phenolics (18 vs. 286 mg CAE/kg, respectively), improved the proteomic coronary artery disease (CAD) score compared with baseline [64]. Positive effects were also seen in another study with healthy subjects, in this case, in a dose-dependent manner, since consumption of an olive oil with high phenolic content (366 mg/kg) decreased systolic blood pressure as compared to low content (2.7 mg/kg) and to pre-intervention values, and it downregulated the expression of genes related to the renin–angiotensin–aldosterone system in peripheral blood mononuclear cells (PBMCs) [65]. Additionally, in an acute intake study, healthy participants ingested functional virgin olive oils (FVOOs) differing in phenolic content (250, 500, and 750 ppm) and in a sustained intake study, hypercholesterolemic participants ingested a control VOO (80 ppm) or FVOO (500 ppm) [66]. Acute and sustained intake of VOO and FVOO resulted in changes associated with diminished atherosclerotic activity as shown by decreased PON1 protein and increased PON1-associated specific activities [67]. Furthermore, mechanistic studies revealed that the intake of isolated phenolic compounds modulated mitogen-activated protein kinases and peroxisome proliferator-activated receptors regulating PON synthesis [66]. With hypercholesterolemic subjects, using 3-week supplementation with VOO (either enriched or not in its own phenolic compounds), 15 HDL-associated differently expressed proteins were found, mainly involved in pathways of LXR/RXR activation, acute phase response, and atherosclerosis [68]. Recently, it was reported that the ingestion of an olive pomace-enriched biscuit (olive pomace being a waste product of olive oil production containing biophenols and fibers (~17 mg/100 g of HT and its derivatives)) by hypercholesterolemic subjects led to increased levels of homovanillic acid and 3,4-dihydroxyphenylacetic acid (possibly involved in reducing oxidative LDL cholesterol) as compared to an isoenergetic control. No statistically significant changes were found in either ox-LDL or urinary isoprostane [69]. In this context, the intake of a virgin oil enriched in phenolic compounds (500 mg/kg) led to an increase in HDL antioxidant compounds in hypercholesterolemic volunteers while increasing the levels of fecal HT and dihydroxyphenylacetic acids [70], as compared with pre-intervention values and a lower-phenolic VOO (80 mg/kg) [71]. Of note, in MS patients the consumption of a high-phenol (398 ppm) VOO-based breakfast, as compared to low (70 ppm) or intermediate (149 ppm) phenol content, limited the increase of postprandial lipopolysaccharide (LPS) plasma levels, and reduced TLR4 and SOCS3 proteins, the activation of NF-κB, and postprandial gene expression of IL6, IL1B, and CXCL1 in PBMCs [60].

With regard to studies where olive oil phenolic compounds were administrated alone, several cardioprotective properties have been reported [72,73,74,75,76,77,78]. In murine models with induced injury or toxicity, treatment with OLE or its aglycone resulted in recurrent features, such as reduction of pro-inflammatory cytokines production (TNF-α and IL-1β), NF-κB expression and translocation, iNOS expression, adhesion molecules, and apoptosis markers, among others [73,74,75]. OLE aglycon has also been reported to interfere with the aggregation of amylin (involved in type-2 diabetes), eliminating its cytotoxicity [79]. Regarding human studies, in patients suffering from ulcerative colitis, OLE-treated colonic samples showed an amelioration of LPS-induced inflammatory damage, accompanied by decreased expression of COX-2 and IL-17 compared to samples exposed to LPS alone [80].

**Figure 1:**
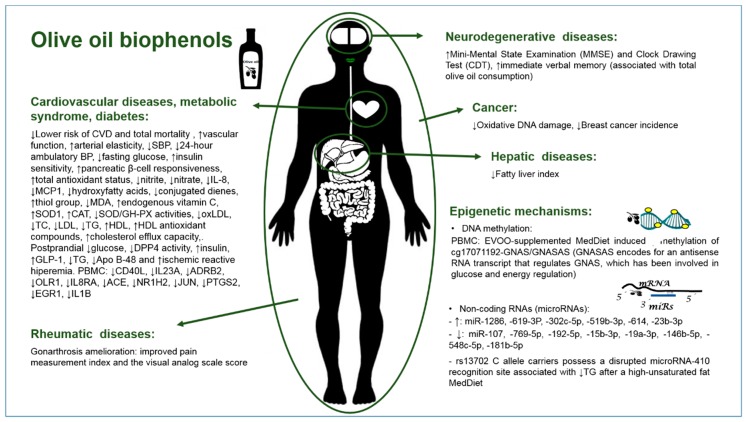
Clinical trials-derived evidence regarding biophenol-rich olive oils’ benefits and mechanisms.

The protective actions of HT, tyrosol (Tyr), and other phenolic compounds present in olive oil against oxidative damage and inflammatory response have been recurrently demonstrated in vitro and in vivo [81]. Recently, in the context of inflammatory response in immune blood cells, pure HT, Tyr, and homovanillic alcohol (HVA) at physiologically relevant concentrations (0.25–1 μM) were able to inhibit oxysterol-induced production of proinflammatory cytokines (IL-1β, MIF, and RANTES), ROS production, and redox-based MAPK phosphorylation (JNK, p38) [82]. In addition, both HT and metabolites (1, 2, 5, and 10 μM) provided protection against endothelial dysfunction in human aortic endothelial cells (HAECs) co-incubated with TNF-α by significantly reducing the secretion of E-selectin, P-selectin, ICAM-1, and VCAM-1, and HT metabolites further reduced levels of monocyte chemoattractant protein 1 (MCP-1) [83]. In TNF-α-treated human umbilical vein endothelial cells (hECs), Tyr and its chemically synthesized metabolites Tyr-glucuronate and Tyr-sulfate (particularly the latter) prevented the phosphorylation of NF-κB signaling proteins. Both metabolites also prevented the over-expression of adhesion molecules and the adhesion of human monocytes to hECs [84]. In addition, Tyr and Tyr-sulfate counteracted TNF-α-induced oxidative stress in these cells and ameliorated edema in mice models of acute and chronic inflammation in a dose-dependent manner. In terms of other phenolic compounds found in VOOs, 3,4-dihydroxyphenylethanol-elenolic acid (3,4-DHPEA-EA) and in particular 3,4-dihydroxyphenylethanol-elenolic acid dialdehyde (3,4-DHPEA-EDA), were shown to significantly protect red blood cells from oxidative damage [85]. In a recent study, MDA levels increased in human endothelial (HECV) cells exposed to a mixture of oleate/palmitate to mimic the condition of atherosclerosis. Treatment with isolated phenolic compounds, apigenin, caffeic acid, coumaric acid, Tyr, and OLE (extracted from olive pomace) significantly decreased MDA levels in these cells. In addition, in these steatotic HECV cells, NO release and NF-κB p65 levels increased significantly with respect to the control. This was counteracted by exposure to phenolic compounds extracted from olive pomace (PEOP) [86]. Regarding recent studies in animal models, in a DSS-induced acute colitis mouse model, hydroxytyrosyl acetate supplementation ameliorated the inflammatory response by modulating cytokine production, along with a reduction in COX-2 and iNOS protein expression, likely through MAPK (p38, JNK) and NF-kB signaling pathways [87]. In a study aiming to assess how HT supplementation differentially affects the adipose and liver tissue proteome, oxidative stress-related proteins were modulated by HT supplementation in both tissues, including a consistent repression of peroxiredoxin 1, which may be indicative of a better antioxidant status [12]. In Wistar rats, both HT- and in particular secoiridoid-supplemented diets (5 mg/kg/day) modulated the aorta and heart proteome compared to the standard diet, downregulating proteins related to proliferation and migration of endothelial cells and occlusion of blood vessels in the former and proteins related to heart failure in the latter [88]. In another study in rats, a high-carbohydrate high-fat diet (MS-inducing diet) + HT (20 mg/kg/day) was effective towards the mobilization of lipids as compared to only an MS-inducing diet, with branched fatty acid esters of hydroxy oleic acids lipids being regulated in the HT-supplemented group, denoting the alleviation of MS [89]. With regard to research in humans, clinical trial-derived evidence where a diet supplemented with phenol-rich olive oils or phenolic extracts is administered is increasing (Table 1, Figure 1). The PREDIMED trial has provided clear proof about the beneficial consequences of a long-term phenol-rich olive oil-supplemented diet in comparison to a low-fat control diet, which are not restricted to cardioprotection [90]. These benefits include improvements in several parameters associated to oxidation, inflammation, hypertension, metabolic syndrome, and diabetes, among others, which translate into lower risk of CVD and total mortality, for instance. Other, recent, short-term (duration of weeks to a few months) and acute studies also support the positive consequences attributed to the consumption of olive oil phenolic compounds (Table 1). Fewer studies in healthy [91] and hyperlipidemic subjects [92] have reported an absence of effect in surrogate markers of CVD, including lipid profile, inflammation, and oxidation, after supplementation with olive oil biophenols.

It should be underscored that the oxidative stress hypothesis is still debated following the null results of antioxidant trials. Therefore, the true contribution of antioxidant actions (unlikely to be direct due to the low bioavailability of biophenols) to cardioprevention is yet to be fully elucidated.

## 5. Neurodegenerative Diseases

Neurodegenerative disorders are age-dependent disorders which are becoming increasingly prevalent, in part because human longevity keeps increasing [113]. These disorders are defined by a multifactorial nature and have common neuropathological hallmarks such as abnormal protein dynamics with defective protein degradation and aggregation, oxidative stress and free radical formation, impaired bioenergetics and mitochondrial dysfunction, and neuroinflammatory and apoptotic processes [114]. Examples of neurodegenerative diseases include AD, Parkinson’s disease, Huntington's disease, and amyotrophic lateral sclerosis, among many others.

Either included in EVOOs or in the form of extracts, administration of phenolic-rich compounds has been demonstrated to exert neuroprotective effects in several in vitro and in vivo studies, as recently reviewed [115]. Olive oil or olive oil extracts containing a mix of phenolic compounds have been demonstrated to counteract age-related dysfunctions in several neuropathology-induced models. The neuroprotective effects seen include the improvement in cognitive behavior and motor coordination, accompanied by a reduction of total Aβ (due to enhanced Aβ clearance pathways and reduced brain production), and tau brain levels, a rise in the activity of detoxifying enzymes, and reduced lipid peroxidation [28,116]. Moreover, in ischemia–reperfusion models, administration of phenolic-rich olive oil reduced infarct volume, brain edema, blood–brain barrier permeability, and improved neurologic deficit scores, as well as brain ceramide levels [117,118]. Furthermore, an olive oil extract (45.5% biophenols, 4.2% HT, 2.2% Tyr, and 9.2% OLE) modulated inflammatory response in LPS-activated astrocytes and serum of multiple sclerosis patients by diminishing MMP-9 and MMP-2 levels and activity [119]. Finally, in amyotrophic lateral sclerosis (ALS) models, in vivo exposure to EVOO phenols resulted in higher survival and better motor performance, with improved muscle status and autophagy markers, and diminished endoplasmic reticulum (ER) stress [120], while in vitro it protected motoneurons from LPS-induced lethality, and inhibited IL-1β and NO release [121].

Concerning studies where pure phenolic-compounds were tested, OC, OLE, HT, and Tyr have been the subject of most research. OC, a naturally occurring phenolic secoiridoid of EVOO, has been attributed several neuroprotective activities. It interacts with relevant actors in different disease-related pathways (ex. inflammation, cancer, neurodegenerative diseases), such as heat-shock proteins (for example by inhibiting Hsp90) [122], and tau-441; this induces stable conformational modifications of the protein secondary structure and also interferes with tau aggregation [123]. This phenolic compound is capable of altering the oligomerization state of Alzheimer’s-associated Aβ oligomers while protecting neurons from their synaptopathological effects [124]. Both in vitro and in vivo, OC was reported to enhance Aβ clearance from the brain via up-regulation of P-glycoprotein and LDL lipoprotein receptor-related protein-1 (major Aβ transport proteins) at the blood–brain barrier [125]. More recently, OC was reported to prevent oligomer (Aβo)-induced synaptic protein SNAP-25 and PSD-95 down-regulation in neurons, and to attenuate Aβo-induced inflammation, glutamine transporter (GLT1), and glucose transporter (GLUT1) down-regulation in astrocytes [126]. In addition, it reduced the Aβo-induced increase of interleukin-6 and glial fibrillary acidic protein (GFAP). As a cautionary note, OC is a high-molecular weight molecule for which bioavailability needs to be ascertained. In addition, the fact that OC crosses the blood–brain barrier remains unproven.

OLE aglycone provided neuroprotection to cultured neuronal cells [127], invertebrate simplified models of Alzheimer’s disease and inclusion body myositis [128], and murine models of amyloid-ß deposition by interfering with Aß aggregation, counteracting the associated neuroinflammation, inducting autophagy, and improving cognitive performance [129,130,131]. Moreover, exposure to OLE protected against apoptosis in murine models of spinal cord injury and cerebral I/R injury, along with reduced infarct volume in the latter [132,133]. Reduced oxidative damage in specific brain areas was also found after OLE administration, as well as increased levels of antioxidant enzymes and improved learning and memory retention [134,135]. Recent studies have supported the protective capacities of HT and Tyr through the reduction in inflammatory markers, downregulation of apoptotic proteins, and ameliorated mitochondrial dysfunction [136,137,138,139]. In this sense, both pre- and post-treatment with HT prevented Aβ(25–35)-induced astrocytic cell line C6 cytotoxicity, induced Akt activation, and reduced the activation of mTOR, leading to improved insulin sensitivity and restoration of proper insulin-signaling [27].

Recent studies suggest that olive oil phenolic compounds are processed by the body as xenobiotics via the Keap1/Nrf2/ARE signaling axis and exert their protective actions through the induction of these enzymes. Yet, no induction of phase II enzymes was found in PBMCs from healthy humans supplemented with HT, and further studies are needed to confirm this hypothesis [91]. In a very recent study using cell-free model assays, EVOO phenolic extracts (rich in secoiridoids derivatives, lignans, and vanillic acid) acted as multi-target ligands directly inhibiting neurodegenerative disorder-related enzymes BuChE, 5-LOX, hMAO-A and hMAO-B in a dose-dependent manner [140].

In summary, in vitro and in vitro neuroprotective activities attributed to olive oil phenolics include interference with amyloid and tau protein aggregation, and reduction of Aβ deposition, production, and induced inflammation, as well as enhanced Aβ clearance, decreased inflammatory biomarkers, oxidative stress, and apoptosis, lessening of cerebral infarct volume and damage after induced injury, and attenuation of insulin resistance, mitochondrial dysfunction, and ATP depletion. On the other hand, human evidence on the neuroprotective actions of olive oil phenolics coming from clinical trials is scarce (Table 1, Figure 1). Of note, the PREDIMED study reported an improvement in Mini-Mental State Examination (MMSE) and Clock Drawing Test (CDT) results, as well as in immediate verbal memory (associated with total olive oil consumption) following long-term consumption of a phenol-rich olive oil-supplemented diet compared to a low-fat control diet [111].

## 6. Hepatic Dysfunction

Continued liver damage can lead to chronic liver diseases, such as simple steatosis and steatohepatitis (steatosis with inflammation and hepatocyte injury and death) and fibrosis, among others, which are highly prevalent worldwide [141]. Accumulating evidence indicates that oxidative stress and inflammation are strongly linked and participate in the pathophysiological processes of liver diseases [44].

Modulation of hepatic lipid metabolism, including protective effects against steatosis [142,143], lipid synthesis [144,145], and endoplasmic reticulum stress [146,147], as well as induction of antioxidant/detoxicant enzymes [148], mitochondrial biogenesis, and mitochondrial function [149] by olive oil and its phenolic compounds has been reviewed recently [150,151]. Recent in vivo studies support a dose-dependent hepatic protective role for olive oil and its phenolic compounds. In C57BL/6J male mice, dietary supplementation with an EVOO (859 mg total biophenols) significantly reduced fat accumulation in liver and the plasmatic metabolic alterations caused by a high-fat diet (HFD) compared to EVOOs with lower amounts (116 and 407 mg) and produced a normalization of oxidative stress-related parameters, desaturase activities, and long-chan polyunsaturated fatty acids (LCPUFA) content in tissues [152]. Moreover, in male Sprague–Dawley rats, a biophenol-rich VOO (0.290 mg phenols/kg/day) was able to (as compared to a phenol-free olive oil), significantly reduce liver inflammation and mitochondrial oxidative stress and restore insulin sensitivity, while limiting HFD-induced insulin resistance, inflammation, and hepatic oxidative stress, preventing nonalcoholic fatty liver disease (NAFLD) progression [153]. Furthermore, the replacement of dietary fat with phenolic-rich EVOO (total phenolic compound concentration: 447 ppm) reversed HFD-induced hepatic steatosis in mice. Also, the use of a phenolics-rich EVOO rather than EVOO (104 ppm) improved the plasma lipid profile and adipose tissue cytokine expression in mice with NAFLD [154]. Olive oil, HT and tyrosol (TY) showed protective effects against TCDD-induced hepatotoxicity in male Wistar rats, restoring ALT, AST, ALP, nitrite, and protein carbonyl content as well as NQO1 and HO. In addition, treatment with olive oil and its phenolic compounds resulted in reduced CYP1A1 and apoptosis (reduction and rise in Bax and Bcl-2 levels, respectively) [155]. In a rat model of NAFLD, the most common chronic liver disease in western countries, HT (10 mg/kg/day) significantly corrected the metabolic impairment induced by HFD, increasing hepatic peroxisome proliferator activated receptor PPAR-α and its downstream-regulated gene fibroblast growth factor 21, the phosphorylation of acetyl-CoA carboxylase [156]. HT also reduced liver nitrosylation of proteins, reactive oxygen species production, and lipid peroxidation. In male mice C57BL/6J, HT supplementation (5 mg/day, for 12 weeks) significantly reduced fat accumulation in liver and plasma as well as tissue metabolic alterations induced by HFD, in addition to a normalization of Δ-5 and Δ-6 desaturase activities and oxidative stress-related parameters as compared to control animals [157]. In Wistar rats, a phenolic-rich olive fruit extract and an OLE extract showed protective effects against deltamethrin-induced hepato-renal toxicity by reducing lipid peroxidation (MDA), Cox-2, and apoptosis (reduction in p53 and rise in bcl-2), and by augmenting total antioxidant capacity and superoxide dismutase (SOD) and catalase (CAT) activities [158]. Treatment with a mix of PEOP was performed on rat hepatoma (FaO) cells exposed to a mixture of oleate/palmitate to mimic the conditions of NAFLD. Tyr, OLE and PEOP significantly reduced the triglyceride (TG) content with respect to steatotic cells. PEOP also decreased the number and size of lipid droplets in steatotic cells as compared to control. Furthermore, exposure to apigenin, caffeic acid, coumaric acid, OLE, and PEOP significantly decreased MDA level in steatotic FaO cells as compared to the control. Uptake of fatty acids (FAs) into hepatocytes and their oxidation are regulated mainly by PPARα, while the anabolic esterification and conversion of FAs to TGs is controlled by PPARγ, for which expression has been shown to increase in NAFLD. Incubation with PEOP resulted in a significant decrease and increase in PPARα and PPARγ expression, respectively, with respect to steatotic cells. With regard to mitochondrial β-oxidation, PEOP led to a further up-regulation of Cpt1 expression with respect to steatotic cells [86]. In male C57BL/6J mice, supplementation with HT attenuated liver metabolic alterations produced by HFD, activating transcription factors PPAR-α and Nrf2, and deactivating NF-κB [159]. Finally, in a recent study where individual compounds were administered, a 21-day dietary supplementation (5 mg/kg bw/day) with OLE or HT maintained higher levels of α-tocopherol in female Wistar rats’ liver compared to a control diet, even though all diets supplied the same daily dose of α-tocopherol [160].

Human evidence on hepatic protective actions of olive oil phenolics coming from clinical trial is scarce and inconclusive (Table 1). Noteworthy, the PREDIMED study reported an improvement in fatty liver index, with potential implications in the delay or slowdown of NAFLD progression [112]. However, other studies where extracts of phenolic compounds from olive oil have been supplemented to healthy and hyperlipidemic subjects have reported an absence of effect on liver function [91,92,97].

## 7. Cancer

Abundant studies offer evidence that oxidative stress, chronic inflammation, and cancer are closely linked. In response to harmful stimulation, such as pathogenic invasion, mechanical injury, and toxicity, the recruitment of inflammatory cells increases the release and accumulation of ROS at the site of damage [161]. This involves the activation of transcription factors, including NF-κB, signal transducer and activator of transcription 3 (STAT3), MAPK, and hypoxia-inducible factor 1α (HIF1α). These transcription factors coordinate the production of inflammatory mediators, including cytokines and chemokines, and COX2, which lead to the recruitment and activation of leukocytes and trigger the same key transcription factors in inflammatory cells, stromal cells, and tumor cells, resulting in more inflammatory mediators being produced and a cancer-related inflammatory microenvironment being generated and propagated [162].

The association between nutrition and oxidative stress may have an important role in cancer and cancer stem cell progression, as well as in therapy [163]. Over the last few years, many in vitro and in vivo studies have demonstrated that olive oil phenolic alcohols and their secoiridoid derivatives possess anticarcinogenic capacities (in many cases not mediated by molecular mechanisms directly related to their anti-oxidant activity) by blocking tumor angiogenesis [164], inhibiting proliferation and invasion [165,166,167,168], inducting apoptosis [169,170], and regulating inflammatory response [171], among others. The molecular mechanisms exerted in vitro and involved in these effects have been recently reviewed. While the exact underlying anticancer molecular mechanisms of OLE, OC, and HT are still not fully known, evidence continues to accumulate. For instance, OC had a notable cytotoxic activity in human melanoma cells but not in normal dermal fibroblasts, accompanied by a significant inhibition of ERK1/2 and AKT phosphorylation and downregulation of Bcl-2 expression [172]. In this sense, not only did OC induce cell growth inhibition more effectively than classical commercially available COX inhibitors, but it also inhibited colony formation and induced apoptosis (PARP cleavage, activation of caspases 3/7, and chromatin condensation) in HCC and CRC cells, whereas it was not toxic to primary normal human hepatocytes. In addition, OC treatment induced DNA damage, increased intracellular ROS production and caused mitochondrial depolarization, in a dose dependent-manner [173]. Finally, OC showed a potential beneficial effect in suppressing growth of hormone-dependent breast cancer and improving sensitivity to tamoxifen treatment [174]. As for OLE, treatment of HepG2 human hepatoma cells inhibited cell viability and induced apoptosis (upregulation of BAX and downregulation of Bcl-2), through activation of the caspase pathway and the modulation of the phosphatidylinositol 3-kinase/protein kinase B (PI3K/AKT) signaling pathway, suppressing the expression of activated AKT [175]. In addition, a combination (compared to separate exposures) of OLE and cisplatin showed enhanced antitumor activity against HepG2, resulting in further elevation of NO content and of the pro-nerve growth factor (NGF)/NGF balance, accompanied by an upregulation of caspase-3 and a downregulation of *MMP*-7 gene expressions, in a dose-dependent manner [176]. Regarding HT, this phenolic compound showed chemopreventive properties by preventing DNA damage in PBMCs and inhibiting (to different extents) proliferation of breast (MDA and MCF-7), prostate (LNCap and PC3), and colon (SW480 and HCT116) cancer cell lines [177]. Moreover, in papillary (TPC-1, FB-2) and follicular (WRO) thyroid cancer cell lines, high doses (with respect to other cancer cells lines) of HT reduced cancer cells viability by promoting apoptotic cell death via an intrinsic pathway [178]. HT and 2HT colonic metabolites (phenylacetic and hydroxyphenylpropionic acid) caused cell cycle arrest and promoted apoptosis in HT-29 and Caco-2 cells [179].

The modulation of the senescence-associated inflammatory phenotype has been suggested to be an important mechanism action of olive oil phenols. Cellular senescence, a process that restricts proliferation of damaged or premalignant cells, also plays a role in aging and age-related diseases, and represents an interesting therapeutic target [180]. In a recent study in pre-senescent human lung (MRC5) and neonatal human dermal (NHDF) fibroblasts, 4–6 weeks of treatment with 1 μM HT or 10 μM OLE aglycone (OLE) reduced β-galactosidase-positive cell number and p16 protein expression, IL-6, metalloprotease secretion, COX-2 and α-smooth-actin levels. In NHDF, OLE and HT treatment counteracted senescence-related rises in COX-2 expression, NF-κB protein level, and nuclear localization. In addition, pre-treatment with these phenolic compounds prevented TNF-α-induced inflammatory effects in these cells [181].

Of note, studies of cancer development and dietary prevention are very difficult to carry out in humans, due to the paucity (or absence) of surrogate markers to be modulated by such interventions. Therefore, even though epidemiological, in vitro, and animal data do suggest chemopreventive effects of olive oil phenolics, this hypothesis might never be confirmed in humans. Nevertheless, studies by Machowetz et al. [108] and Salvini et al. [107] in healthy males and postmenopausal women, respectively, have reported reduced oxidative DNA damage after short-term ingestion of phenol-rich olive oil. More recently, the PREDIMED trial reported a diminution in the incidence of breast cancer following long-term consumption of a phenol-rich olive oil-supplemented Mediterranean diet as compared to a low-fat control diet [22].

## 8. Rheumatic Diseases

There are more than 200 different conditions that are labelled as rheumatic diseases, including rheumatic arthritis, systemic lupus erythematosus, and osteoarthritis (OA), among others. One of the major characteristics of rheumatic diseases is chronic inflammation and autoimmunity, which consequently leads to tissue destruction and reduces patient mobility [182]. Immune cells play a key role in inflammation due to involvement in initiation and maintenance of the chronic inflammatory stages. In particular, circulating monocytes that may differentiate towards macrophages or dendritic cells are able to produce proinflammatory cytokines and mediators (including ROS and COX-2), attracting T and B cells which contribute to maintaining the inflammatory process and eventually to tissue destruction.

Several in vitro and in vivo studies have been carried out with models of chronic inflammation and autoimmunity exposed to olive oil phenolics. LPS-exposed J774A.1 macrophages treated with olive oil biophenol extracts showed reduced iNOS and COX-2 expression (100 μg phenols/mL), and NO release in a dose-dependent manner (50–150 μg/mL) [183]. Furthermore, OC repressed MIP-1α, IL-6, IL-1β, and TNF-α levels, as well as GM-CSF protein synthesis and LPS-induced NO production in this cell line [184]. In a collagen-induced arthritis mice model, an EVOO biophenol extract significantly reduced the levels of proinflammatory cytokines, COX-2, and microsomal prostaglandin E synthase-1, inhibiting c-Jun N-terminal kinase, p38 and STAT-3, and reducing NF-κB translocation [185]. In the same mice model, intake of a HT acetate-supplemented diet significantly prevented arthritis development and decreased serum IgG1 and IgG2a, cartilage olimeric matrix protein (COMP) and metalloproteinase-3 (MMP-3) levels, as well as pro-inflammatory cytokine levels (TNF-α, IFN-γ, IL-1β, IL-6, and IL-17A). The activation of JAK/STAT, MAPKs, and NF-κB pathways were drastically ameliorated, whereas Nrf2 and HO-1 protein expressions were significantly up-regulated [186]. In male Wistar rats with induced rheumatoid arthritis, supplementation with HT-enriched refined olive oil led to decreased histological damage, as well as reduced COX-2 and iNOS expression [187]. OA progression is characterized by increased NO production, which has been associated with cartilage degradation. OC and its derivatives decreased MIP-1α and IL-6 levels [184], as well as lipopolysaccharide-induced NO synthase (NOS2) synthesis in ATDC-5 chondrocytes [188]. Although a consensus on the actual role of autophagy in OA has not been reached, several studies showed it is decreased in OA, and its activation is protective against OA [189]. HT increased markers of autophagy and protected human C-28/I2 and primary OA chondrocytes exposed to hydrogen peroxide from DNA damage and cell death induced by oxidative stress. This autophagy-inducing effect is engaged through SIRT1-dependent and -independent mechanisms [190]. In a pristane-induced systemic lupus erythematosus (SLE) mice model, administration of EVOO containing high levels of phenolic compounds (600 ppm) reduced renal damage and MMP-3 serum and PGE2 levels in the kidney, as well as proinflammatory cytokine production in splenocytes, while up-regulating Nrf-2 and HO-1 protein expression and the activation of JAK/STAT, MAPK, and NF-κB pathways [191]. Moreover, in PBMCs from patients with SLE and healthy donors, the phenolic fraction of EVOO modulated cytokine production (IFN-γ, TNF-α, IL-6, IL-1β, and IL-10) and attenuated induced T-cell activation, possibly via NF-κB signaling pathway, as increased expression of I-kappa-B-α and decreased extracellular signal regulated kinase phosphorylation accompanied these anti-inflammatory and immunomodulatory regulations [192].

To date, very few human studies (to the best of our knowledge) have been performed to ascertain the potential pharma-nutritional activity of olive biophenols in rheumatic disorders. Conceivably, their anti-inflammatory properties should augment the habitual pharmacological therapy of such diseases and contribute to increase patient wellbeing. In this context, supplementation of a HT extract to early-stage knee OA subjects for 4 weeks improved the pain measurement index and the visual analog scale score [109].

### Epigenetic Studies

Epigenetics is the study of heritable variations in gene function that cannot be attributed to changes in the sequences of coding DNA. There are causal interactions between genes and their products that give rise to the phenotype. In terms of lifestyle, it is noteworthy that different diets providing, e.g., different fatty acids [193] can modulate genetics through epigenetic changes. Several investigators reported epigenetic variations through the study of the mechanisms by which dietary exposure can have long-term consequences for growth and health. As an example, Mathers et al. developed a model of four Rs (Received’, ‘Recorded’, ‘Remembered’, and ‘Revealed’) to explain the mechanism of nutritional epigenomics [194]. Other publications addressed the issue of how diet in pregnancy affects fetal programming [195].

All epigenetic variations are most often investigated by assessing histone modification, DNA methylation, and non-coding RNAs. Histone modifications by methylation, acetylation, ubiquitination, and phosphorylation determine an active or inactive state of chromatin and, thus regulate gene expression. DNA methylation consists of the addition of methyl groups at the 5-position of a cytosine and is frequently part of a cytosine-guanine dinucleotide (CpG). These are clustered in the 5′ ends of genes in regions known as “CpG islands.” This methylation is associated with the silencing of gene transcription and is a dynamic process that occurs throughout life [196]. Table 2 includes studies reporting epigenetic changes induced by olive oil (OO) through histone modification and DNA methylation mechanisms. Finally, non-coding RNAs are not translated into a protein, but are transcribed from DNA. They participate—in various forms—in the regulation of gene expression. There are different types of non-coding RNAs, but in this paper we focused on studies where the modulation of microRNAs (miRs) by olive oil and its phenolic components was assessed. MiRs, about 18–25 nucleotides in length, were identified for the first time in 2001 by Lagos-Quintana et al. [197]. The function and biogenesis of miRs has been predicted by *lin-4* and *let-7*, which were firstly identified by genetic analyses of *Caenorhabditis elegans* [198,199]. Developmental timing is generated in the cell nucleus as immature particles (pri-miRNA), which are recognized by the nuclear protein DGCR8, associated with the enzyme Drosha to release hairpins from pri-miRNAs and produce the pre-miRNAs. Pre-miRNA hairpins are exported by exportin-5 to the cytoplasm, where the RNase III enzyme Dicer interacts with the 3′ end of the hairpin and cleaves the loop joining the 3′ and 5′ arms. Finally, two strands are generated, one that is incorporated into the RISC complex and another that is degraded. After being processed, miRs act principally as transcriptional repressors of mRNA expression [200,201]. MiRs do not need to be totally complementary to their seed region of mRNAs; therefore, the alteration of a single micro-RNA can change the expression of multiple genes [202]. For this reason, the regulation of miRs through diet or through pharma-nutritional interventions is being proposed as a valuable therapeutic strategy in various diseases, because it would modulate functionally-related pathway genes via epigenetic changes. The literature reports many changes in miR profiles induced by the consumption of different types of OO, namely EVOO. In animal model studies, aged mice were treated with extra-virgin olive oil rich in phenols (6 mg/kg) for six months, and miR modulation in brain tissue was observed; such modulation appears to exert positive regulatory effects on neuronal function [203]. Epigenetic investigations were performed in pregnant Sprague–Dawley rats fed with different oils, i.e., soybean oil (SO), OO, fish oil (FO), linseed oil (LO), or palm oil (PO), from conception to day 12 of gestation and with a standard diet thereafter. MiRs expression was assessed in the liver and in adipose tissue. The results show that maternal consumption of different types of oils influences miR expression and may epigenetically explain the long-term phenotypic changes of the offspring [204]. Regarding human studies (Figure 1), we found two studies in which researchers analyzed the epigenetic changes (through miR assessment) occurring after OO consumption. The interaction of an miR target site SNP with diet and its effects on triglycerides and stroke is one of the many studied outcomes of the PREDIMED trial. In this study, 7187 participants were assigned to three groups: (1) low-fat diet (control); (2) EVOO- or (3) nut-supplemented Mediterranean diet. Researchers found that miR-410 regulated lipoprotein lipase variant rs13702, which is associated with stroke incidence and controlled by diet [205]. Another human research study addressing the effects of supplementations with acute high- and low-phenols EVOO intake on miRs expression was performed on PBMCs of healthy subjects and patients with metabolic syndrome (MS). The result indicated that high-biophenols EVOO intake is able to modify the miR profile; these potentially relevant effects are stronger in healthy subjects [206].

Specific to OO phenolics, some studies analyzed epigenetic changes in miRs produced by HT and/or OLE. Studies in cell cultures with OLE at 200 μM (i.e., non-physiological concentrations) noted that human NPC cell lines and a xenograft mouse model, both irradiated, underwent strongly enhanced radiosensitivity via reduction of the activity of the HIF1α-miR–519d–PDRG1 pathway, which is essential to radiosensitization [207]. In a study where human ovarian cancer cell lines were used for xenograft assay and were irradiated and treated with 200 µM of OLE, the treatment altered the miR expression profile, specifically; the endogenous expression of miR-299 was repressed by a hypoxia inducible factor and reassured with OLE treatment [208].

To the best of our knowledge, there are no studies that report modulation of miRs by Tyr. Conversely, two papers addressed the actions of HT. In one study, HT modulated the expression of several miRs. In mice supplemented with nutritionally relevant amounts of HT (0.03 g), for eight weeks, changes were found in the expression of miRs in the intestines. The analysis of other tissues revealed consistent HT-induced modulation of only few miRs, e.g., miR-483. In vitro mechanistic studies that used treatment with HT at 10 μM of a human colonic adenocarcinoma cell line (Caco-2), human primary epithelial intestinal cells (InEpCells), and mouse primary organoids confirmed modulation of these miRs. Lastly, one miRNA, miR-193a, was modulated in healthy volunteers supplemented with HT for one week [209]. In a study aimed at elucidating the mechanisms via which OO biophenols modulate miRs, HT, but not OLE (both at 10 μM), induced NRf2 nuclear translocation and reduced miR-146a expression in macrophage RAW 264.7 cells with induced inflammation [210]. Taken together, these studies suggest that both EVOO and its phenolic compounds, together or separately, have effects on the modulation of miRs. In other words, the use of EVOO as principal source of fat modulates our genes through epigenetic changes. Before solid conclusions can be drawn, we would like to underscore that this is a very broad field of research, in which many more studies need to be done. For example, the use of long-term generational research will eventually uncover the true effect of epigenetic changes reported thus far. In addition, future studies will elucidate the possible beneficial effects attributed to the moderate consumption of EVOO in terms of nutrigenomic and epigenetic consequences.

## 9. Conclusions

Nutrition science is shifting focus from caloric intake and macronutrient proportions to the molecular, “pharmacological” actions of food components. Pharma-nutrition partly helps overcome the many hurdles that impede providing sound dietary advice [217]. As many general reviews on olive biophenols are available [18], in this paper we focused on recent evidence (published in the last decade) of the cellular and molecular actions of these interesting molecules. Accumulated data do indicate that olive biophenols, chiefly hydroxytyrosol, have properties that largely explain the cardioprotective effects of diets where EVOO is the most prominent added fat [218]. It should be underscored that evidence-based pharmacology would require several high-quality human trials before health claims can be exhibited [219]. With regard to olive oil and its biophenols, these studies are urgently needed if we want to substantiate the numerous biological properties of these compounds. However, this is very difficult to implement in the area of nutrition [7]. Therefore, caution should be exerted before the formulation of strong, definitive statements about olive oil and its components, and as a matter of fact, any food ingredient. However, it is worth noting that the available evidence on olive biophenols is abundant and scientifically allows suggesting the use of high-quality olive oil as the principal form of dietary fat. Whether isolated molecules or well-characterized extracts could be employed as pharma-nutritional adjunct agents to, e.g., lessen inflammation and improve prognosis of inflammatory diseases should be addressed by future, high-quality human studies.

## Figures and Tables

**Table 1:** Randomized clinical trials-based evidence on the effects and mechanisms after the consumption (acute or sustained) of phenol-rich olive oil and olive oil phenolic extracts.

Cardiovascular Disease, Metabolic Syndrome, T2DM						
Subjects	Extract/OO	Duration	OO Phenolic Content Treatment (Daily)	OO Phenolic Content Control (Daily)	Main Results vs. Control	Reference
Healthy males	OO	3 week	8.38 or 3.76 mg TP	0.06 mg TP	Phenolic dose-dependent ↓oxLDL, ↑HDL and ↓TC/HDL	[93]
Healthy men	OO	3 week	14.4 mg TP	0 mg	↓oxLDL, ↓hydroxy fatty acids, ↓conjugated dienes	[94]
Metabolic syndrome	OO	Once	14.5 mg TP	2.56 mg TP	Postprandial ↓JUN, ↓PTGS2, ↓EGR1, ↓IL1β in PBMC	[95]
Healthy adults	OO	3 week	8.38 mg TP	0.06 mg TP	↓oxLDL, ↓MCP1. PBMCs: ↓CD40L, ↓IL23A, ↓ADRB2, ↓OLR1, ↓IL8RA	[96]
Overweight men	Extract	12 week	51.1 mg OLE/9.7 mg HT	0 mg	↑Insulin sensitivity, pancreatic β-cell responsiveness	[97]
Healthy elderly	OO	6 week	EVOO as the only diet-added fat, +24.5 mg TP	unspecified, control group maintained dietary habits	↑TAC, ↑CAT, ↓SOD and GH-PX activity, ↓LDL, ↓TG, ↑HDL	[98]
Healthy males	OO	3 week	8.38 mg TP	0.06 mg TP	↑Cholesterol efflux capacity	[99]
High cardiovascular risk	OO	1 year	EVOO (≥50 g, unspecified TP)-supplemented Mediterranean diet	unspecified, control group discouraged to consume olive oil	↓24-h ambulatory blood pressure (BP), ↓TC, ↓fasting glucose	[100]
Healthy adults	Extract	Once	51 mg OLE/10 mg HT	0 mg	↑Vascular function, ↓IL-8	[101]
Hypercholesterolemic	OO	3 week	11.45 mg TP	1.83 mg TP	↑Proteins related to cholesterol homeostasis, protection against oxidation and blood coagulation, ↓proteins implicated in acute-phase response, lipid transport, and immune response	[68]
Postmenopausal women with osteopenia	Extract	1 year	~120 mg TP	0 mg	↓TC, ↓LDL, ↓TG	[102]
Pre- and hypertensive adults	OO	Once	26.41 mg TP	7.94 mg TP	Postprandial ↓oxLDL, ↑ischemic reactive hyperemia	[103]
Healthy	Extract	1 week	5 or 25 mg HT	0 mg	No effect on lipid profile, inflammation, and oxidation markers	[91]
Arterial stiffness risk	Extract	11 days	50 or 100 mg HT	0 mg	↑Arterial elasticity, ↓TG	[104]
Mild hyperlipidemic	Extract	8 week	45 mg HT	no control	vs. baseline: ↑endogenous vitamin C; no influence on markers of CVD, blood lipids, inflammatory markers	[92]
Healthy adults	OO	Once	4.35 mg TP	0 mg	Postprandial ↓glucose, ↓DPP4 activity, ↑insulin, ↑GLP-1, ↓TG, ↓Apo B-48	[105]
Healthy males	OO	3 week	8.38 mg TP	0.06 mg TP	↓SBP. PBMC: ↓ACE, ↓NR1H2, ↓IL8RA	[65]
High cardiovascular risk	OO	~4.8 years	same as [100]	same as [100]	↓Lower risk of CVD and total mortality in elderly independently associated with high urinary HVA (HT metabolite)	[90]
Healthy adults	Extract	3 week	15 mg HT	0 mg	↑Thiol group, ↑TAS, ↑SOD1, ↓nitrite, ↓nitrate, ↓MDA	[106]
Hypercholesterolemic adults	OO	3 week	26.41 mg TP	7.94 mg TP	↑HDL antioxidant compounds	[70]
**Cancer**						
Postmenopausal women	OO	8 week	29.6 mg TP	7.35 mg TP	↓Oxidative DNA damage	[107]
Healthy males	OO	3 week	8.38 or 3.76 mg TP	0.06 mg TP	↓Oxidative DNA damage (phenolic content-independent)	[108]
High cardiovascular risk	OO	~4.8 years	same as [100]	same as [100]	↓Breast cancer incidence	[22]
**Rheumatic diseases**						
Early-stage knee osteoarthritis	Extract	4 week	10.04 mg HT	0 mg	Improved pain measurement index and visual analog scale score	[109]
**Neurodegenerative diseases**						
High cardiovascular risk	OO	~4.8 years	same as [100]	same as [100]	↑Immediate verbal memory (associated with total OO consumption)	[110]
High cardiovascular risk	OO	6.5 years	same as [100]	same as [100]	↑Mini-Mental State Examination and Clock Drawing Test	[111]
**Hepatic Dysfunction**						
Overweight men	Extract	12 week	51.1 mg OLE/9.7 mg HT	0 mg	No effect on markers of liver function	[97]
Healthy	Extract	1 week	5 or 25 mg HT	0 mg	No effect on markers of liver function	[91]
Mild hyperlipidemic	Extract	8 week	45 mg HT	no control	No effect on markers of liver function	[92]
High cardiovascular risk	OO	6 years	same as [100]	same as [100]	↓Fatty liver index	[112]

OO, olive oil; TP, total phenols; HT, hydroxytyrosol; OLE, oleuropein; CVD, cardiovascular disease; TAS, total antioxidant status; TAC, total antioxidant capacity; SOD, superoxide dismutase; MDA, malondialdehyde; HVA, homovanillyl alcohol; HDL, high density lipoproteins; ox-LDL, oxidized low density lipoproteins; TG, triglycerides; TC, total cholesterol; PBMC, peripheral blood mononuclear cell; CAT, catalase; JUN, Jun proto-oncogene, AP-1 transcription factor subunit; PTGS2, prostaglandin-endoperoxide synthase 2; EGR1, early growth response protein 1; IL, interleukin; MCP1, monocyte chemoattractant protein 1; CD40L, CD40 ligand, ADRB2, adrenoceptor Beta 2; OLR1, oxidized low-density lipoprotein receptor 1, GH-PX, glutathione peroxidase; DPP4, dipeptidyl peptidase-4; GLP-1, glucagon-like peptide 1; Apo B-48, apolipoprotein B-48; ACE, angiotensin-converting enzyme; NR1H2, nuclear receptor subfamily 1 group H member 2; EVOO, extra virgin olive oil; T2DM: type 2 diabetes.

**Table 2:** Epigenetic studies on olive oil and its biophenols.

Dietary Component	Doses	Model	Epigenetic Study	Result	Ref.
**DOA**	5, 10, and 20 μmol/L	HMLER cells Female athymic nude mice with SUM-159 cells (Tumor)	DNA methylation	DOA’s ability to strongly and negatively impact the tumorigenic and self-renewal nature of cancer stem cells occurs through DNA methyltransferase -related epigenetic regulation.	[211]
**MedDiet + EVOO**	(1 L/week)	-Human	DNA methylation	Methylation changes in several peripheral white blood cell genes.	[212]
**CO, OO or SO**	CO: 80% OO: 15% SO: 12%	Sprague–Dawley rats -3T3-L1	DNA methylation	Methylation levels changes of the CpG island at the Vegfb promoter and in the Vegfb expression levels in vivo and in vitro by different dietary fatty acids.	[213]
**LCO, HCO or EVOO**	LCO: 3% HCO: 20% EVOO: 17% *w*/*w*	Sprague–Dawley rats	DNA methylation & histone modifications	EVOO diet increased the levels of DNA methylation in mammary glands and tumor and changed histone modifications patterns. CO diet increased DNA methyltransferase activity in both tissues, resulting in an increase in the promoter methylation of the tumor suppressor genes *RASSF1A* and *TIMP3*.	[214]
**EVOO**	100 ppm EVOO 250 μL/300 g	-Caco-2 cells Sprague–Dawley rats	DNA methylation	In vivo and in vitro evidence that DNA methylation of CB1, already associated with a cancer phenotype, can be modulated by EVOO.	[215]
**OLE**	100 μM	aged TgCRND8 mice	Histone modifications	OLE activates neuronal autophagy; it increases histone 3 and 4 acetylation, decreases histone deacetylase 2 expression, and causes a significant improvement in synaptic function.	[130]
***n*-3 LCPUFA or OO**	4 g daily	PBMCs from men and women	DNA methylation	*n*-3LCPUFA or OO can induce selective changes in the methylation status of individual CpG loci in specific genes, which is contingent on the sex of the subject and the nature of the supplement.	[216]

DOA: decarboxymethyl oleuropein aglycone; MedDiet: mediterranean diet; EVOO: extra virgin olive oil. CO: coconut oil; OO: olive oil; SO: sunflower oil; LCO: low corn oil; HCO: high corn-oil; OLE: oleuropein aglycone; *n*-3 LCPUFA: *n*-3 long-chain polyunsaturated fatty acids; PBMCs: peripheral blood mononuclear cells.
